# Factors Associated with Multimodal Care Practices for Cancer Cachexia among Pharmacists

**DOI:** 10.3390/curroncol31100457

**Published:** 2024-10-12

**Authors:** Satomi Okamura, Koji Amano, Saori Koshimoto, Sayaka Arakawa, Hiroto Ishiki, Eriko Satomi, Tatsuya Morita, Takashi Takeuchi, Naoharu Mori, Tomomi Yamada

**Affiliations:** 1 Department of Medical Innovation, Osaka University Hospital, 2-2 Yamadaoka, Suita, Osaka 565-0871, Japan; satomi.okamura@dmi.med.osaka-u.ac.jp (S.O.); tomomi.yamada@dmi.med.osaka-u.ac.jp (T.Y.); 2 Department of Supportive and Palliative Care, Osaka International Cancer Institute, 3-1-69 Otemae, Chuo-ku, Osaka 541-8567, Japan; 3 School of Health Care Sciences, Faculty of Medicine, Institute of Science Tokyo, 1-5-45 Yushima, Bunkyo-ku, Tokyo 113-8519, Japan; skoshimoto-rd@umin.ac.jp; 4 Faculty of Human Nutrition, Department of Human Nutrition, Tokyo Kasei Gakuin University, 22 Sanban-cho, Chiyoda-ku, Tokyo 102-8341, Japan; 5 Department of Palliative Medicine, National Cancer Center Hospital, 5-1-1 Tsukiji, Chuo-ku, Tokyo 104-0045, Japan; sarakawa@ncc.go.jp (S.A.); hishiki@ncc.go.jp (H.I.); esatomi@ncc.go.jp (E.S.); 6 Palliative and Supportive Care Division, Seirei Mikatahara General Hospital, 3453 Mikatahara-cho, Kita-ku, Hamamatsu 433-8558, Shizuoka, Japan; tmorita@sis.seirei.or.jp; 7 Liaison Psychiatry and Psycho-Oncology Unit, Department of Psychiatry and Behavioral Sciences Graduate School of Medical and Dental Sciences, Institute of Science Tokyo, 1-5-45 Yushima, Bunkyo-ku, Tokyo 113-8510, Japan; okaspsyc@tmd.ac.jp; 8 Department of Palliative and Supportive Medicine, Graduate School of Medicine, Aichi Medical University, 1-1 Yazakokarimata, Nagakute 480-1195, Aichi, Japan; nmori@aichi-med-u.ac.jp

**Keywords:** pharmacist, multimodal care, cancer cachexia, knowledge, practice

## Abstract

Pharmacists’ roles in cachexia care are unclear. This study aimed to clarify the knowledge and practice of cachexia care and identify factors related to the practice of cachexia care among pharmacists. Information on the knowledge and practice of cachexia care was obtained. Components of practicing multimodal care were evaluated. Participants were categorized into two groups according to practicing multimodal care levels. Comparisons were made between the groups, and multiple regression analysis was employed. Of the 451 pharmacists, 243 responded. They were categorized into the Practicing group (n = 119) and Not practicing group (n = 124). Significant differences were observed for the number of advanced cancer patients/month, frequency of caring for them, and involvement in training programs on cachexia. The Practicing group had significantly better knowledge about cachexia. The Practicing group used guidelines, items, and symptoms more frequently to detect cachexia. The Practicing group tended to detect cachexia and initiate interventions in earlier phases and in patients with a better status. Multivariate logistic regression analysis showed that the most significant factor was the regular provision of care (odds ratio, 2.07; 95% confidence interval, 1.10–3.92). The regular provision of care was associated with the practice of multimodal care.

## 1. Introduction

Cancer cachexia is considered a wasting disease characterized by imbalances in protein and energy metabolism caused by reduced dietary intake and systemic inflammation [1,2]. Cancer cachexia cannot be completely reversed by conventional nutritional care and can cause physical and psychological symptoms, as well as emotional distress, in patients with advanced cancer [1,2,3,4]. Thus, their daily lives (e.g., eating, activity, and sleeping habits) are severely disrupted [3,4]. 

Several studies and guidelines have been published on the care of patients with cancer cachexia [5,6,7]. According to these guidelines, nutritional intervention, pharmacological intervention, and psychological support for the patient and the patient’s family caregiver are recommended [5,6,7]. Furthermore, the importance of multimodal care (i.e., multidisciplinary care by multiple professionals) in the implementation of these interventions is emphasized [5,6,7]. Therefore, it is necessary that not only the primary physician and nurses but also palliative care physicians, palliative care nurses, pharmacists, dietitians, physical therapists, psychologists, and social workers cooperate to care for patients with cancer cachexia and their family caregivers, using a multidisciplinary perspective [8].

In the practice of multimodal care for cancer cachexia, pharmacists have to contribute to the pharmacotherapy of cancer treatment and palliative care to provide safe and effective pharmacotherapy to patients [9,10]. Specific duties of pharmacists in cancer care include understanding pharmacotherapy, providing information to clinicians and patients, monitoring treatment, and identifying and dealing with adverse events [11,12]. Pharmacists are expected to play a role as pharmacological experts and have a high level of knowledge and skills in pharmacotherapy, as well as the ability to practice it [13,14]. 

However, there have been few studies that focused on the role of pharmacists in multimodal care for cancer cachexia [8,9,10,15,16,17]. Notably, Amano et al. conducted a nationwide survey on the perceptions and clinical practice of clinicians regarding multimodal care for cancer cachexia in Japan [18]. In the present study, we investigated pharmacists’ perception and practice of cancer cachexia care using the previous survey data. The first aim of this study was to clarify the knowledge, perception, and practice of cancer cachexia care among pharmacists involved in cancer care, and the second aim was to identify factors related to their practice of cancer cachexia care.

## 2. Methods

This study employed a preplanned secondary analysis of a nationwide survey conducted among clinicians to understand their perceptions of multimodal care for cancer cachexia. The methodology was reported previously [18]. In brief, an anonymized self-report questionnaire was administered to 451 cancer-designated hospitals across Japan from February to March 2022. The eligibility criteria were clinicians who had 3 years or more of clinical practice experience and those who were engaging in cancer care. One representative physician, nurse, pharmacist, and dietitian from each of the participating facilities were asked to complete the questionnaire. In this study, only survey data obtained from pharmacists were analyzed. Advanced cancer was defined in the questionnaire as locally advanced cancer, metastatic cancer, and hematologic neoplasms.

### 2.1. Ethics

The invitation letter informed potential participants that the survey was anonymized, and that the data would be confidentially analyzed. The completion and return of the questionnaire were considered as consent to take part in the survey. If participants refused to participate in the survey, they were requested to return the questionnaire with “no participation” indicated. If participants did not meet the eligibility requirements, they were requested to return the questionnaire with “not eligible” indicated. 

Since the survey was only conducted among clinicians, the study involved minimal risk and was out of the scope of the Ethical Guidelines for Medical and Health Research Involving Human Subjects in Japan, and national policies did not require approval from the Institutional Review Board of the National Cancer Center (Institute Research Number: 6000-050) [19]. 

### 2.2. Questionnaire

The self-report questionnaire consisted of the following five major sections: (a) participant characteristics, (b) knowledge and application of the international definition and clinical practice guidelines, (c) perception and knowledge of cancer cachexia, (d) items and symptoms used in the assessment of cancer cachexia, and (e) beliefs and perceptions of multimodal care for cancer cachexia.

(a) Participant Characteristics

We obtained data on age, sex, practicing experience, practicing experience of cancer care, number of patients with advanced cancer per month, primary area of practice, frequency of caring for patients with advanced cancer, and involvement in training programs on the management of cancer cachexia.

(b) Knowledge and Application of International Definition and Clinical Practice Guidelines

We collected information about participants’ knowledge and application of the international definition advocated by Fearon et al. [1] and clinical practice guidelines of the American Society of Clinical Oncology (ASCO) [5], European Society for Medical Oncology (ESMO) [6], and European Society of Clinical Nutrition and Metabolism (ESPEN) [7] using yes or no questions.

(c) Perception and Knowledge of Cancer Cachexia

We surveyed the status regarded as cancer cachexia, such as weight loss rate over 6 months, the Eastern Cooperative Oncology Group Performance Status [ECOG PS] [20], and life expectancy. We asked participants to select one answer for each question regarding weight loss rate, ECOG PS, and life expectancy. Answers that indicated the need for nutritional and physical interventions were also considered in each question concerning weight loss rate, ECOG PS, and life expectancy.

(d) Items and Symptoms Used in the Assessment of Cancer Cachexia

The participants evaluated a total of 8 items: dietary intake, physical function, blood tests, computed tomography or magnetic resonance imaging, body weight or body mass index, triceps skinfold thickness or arm circumference, bioelectrical impedance analysis, and double X-ray absorptiometry. They also assessed 15 symptoms: fever, pain, nausea and vomiting, taste and smell changes, lack of appetite, early satiety, reduced food intake, weight loss, fatigue, impaired physical function, drowsiness, depression, anxiety, sleep disorder, and impaired orientation.

(e) Beliefs and Perceptions of Multimodal Care for Cancer Cachexia

The participants assessed a total of 9 components about beliefs and perceptions regarding multimodal care for cancer cachexia, which were previously extracted through a literature review [4]: management of physical symptoms, management of psychological symptoms of illness, provision of nutritional and exercise interventions according to clinical practice guidelines, enablement of adherence to multimodal therapies, aid for the emotional adaptation of patients and family members, support for coping by patients and family members, provision of evidence-based information on cancer cachexia to patients and family members, education on cancer cachexia for patients and family members, and end-of-life discussions with patients and family members. The participants evaluated the 9 components using the following 7-point Likert scale: (1) strongly disagree, (2) disagree, (3) somewhat disagree, (4) neither agree nor disagree, (5) somewhat agree, (6) agree, and (7) strongly agree. 

### 2.3. Statistical Analysis

Of all the participants in the survey, the statistical analyses were performed exclusively for the pharmacists in this study. Eligible pharmacists whose total scores for the 9 components were greater than the median were categorized into the group practicing multimodal cachexia care (Practicing group), and the other pharmacists were categorized into the group not practicing multimodal cachexia care (Not practicing group). 

All comparisons between the two groups were set at the two-sided significance level of 0.05. For participant characteristics; knowledge and application of the international definition and clinical practice guidelines; perception and knowledge of cancer cachexia; and the numbers of guidelines, items, and symptoms used in the assessment of cancer cachexia, Student’s t-test was used for continuous variables, and Fisher’s exact test and the chi-square test (depending on the number in each cell) were used for categorical variables. 

In the search for factors associated with practicing multimodal care for cancer cachexia, multivariate logistic regression analysis was performed. In the logistic regression model, the variables with statistical significance in the group comparisons were included. This method was used because all the variables were clinically meaningful, and the purpose of the analysis was to simply determine factors associated with practicing multimodal care. 

All statistical analyses were performed using SAS version 9.4 software (SAS Institute, Inc., Cary, NC, USA).

## 3. Results

Of the 451 pharmacists who were contacted, 243 responded (53.9% response rate). Among all participating pharmacists, the median (interquartile range) score regarding the nine components of multimodal care for cancer cachexia was 32 (25–38). As a result, 119 pharmacists were categorized into the Practicing group, and 124 pharmacists were categorized into the Not practicing group. 

**Figure 1:**
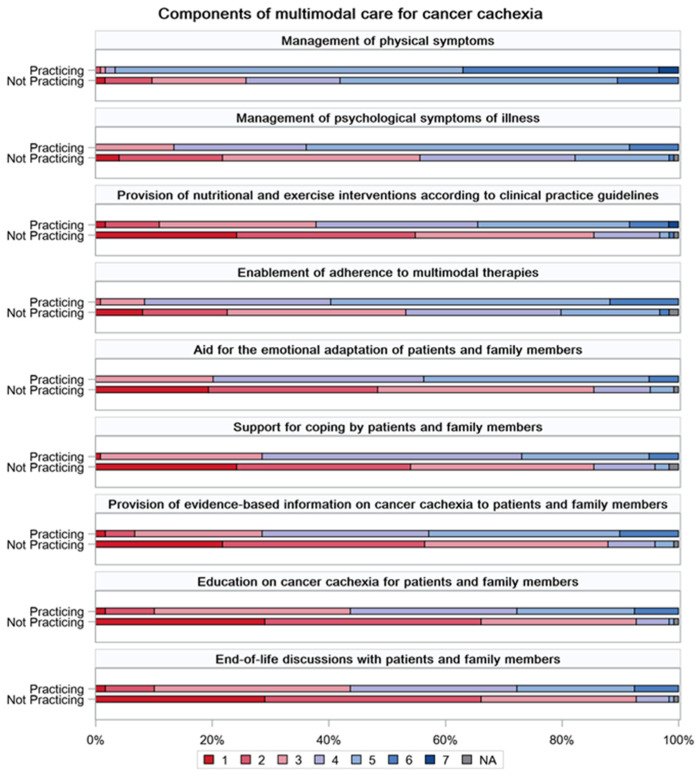
Practicing multimodal care for cancer cachexia. Nine questions about beliefs and perceptions of multimodal care for cancer cachexia were assessed by the participating pharmacists using the following seven-point Likert scale: (1) strongly disagree, (2) disagree, (3) somewhat disagree, (4) neither agree nor disagree, (5) somewhat agree, (6) agree, and (7) strongly agree. Percentages of each point in the group practicing multimodal cachexia care and the group not practicing multimodal cachexia care are shown in the bar graphs. NA, not applicable.

Regarding the practice of multimodal care for cancer cachexia, percentages of the scores for the nine components in the Practicing and Not practicing groups are illustrated using a bar graph in Figure 1. For all components, the Practicing group had a higher perception of the practice.

Participant characteristics are shown in Table 1. The average age was 40.0 years old, and there were slightly more males (56.0%) than females (44.0%). Furthermore, 50.2% of the participants had 10 or more years of practicing experience in cancer care, and 51.4% were involved in the treatment of 20 or more patients with advanced cancer per month. The most frequent primary area of practice was cancer treatment, followed by palliative care and others. Regarding the frequency of caring for patients with advanced cancer, 59.7% of the participants answered “regularly,” whereas 38.7% of them answered “only the first time” or “when needed.” The percentage of pharmacists who had participated in training programs on the management of cancer cachexia was only 7.4%. 

Comparing the Practicing and Not practicing groups in terms of characteristics, significant differences were observed in the number of patients with advanced cancer per month, frequency of caring for patients with advanced cancer, and involvement in training programs on the management of cancer cachexia. The Practicing group tended to care for more patients with advanced cancer per month and were more regularly engaging in caring for them. Additionally, the Practicing group had more opportunities to participate in training programs on the management of cancer cachexia.

Proportions of knowledge and application of the international definition and clinical practice guidelines are presented in Table 2. The Practicing group showed higher proportions in all items. However, less than half of the pharmacists knew the ASCO, ESMO, and ESPEN guidelines, even in the Practicing group. 

Proportions of perception and knowledge of cancer cachexia are shown in Table 3. Concerning both weight loss rate and ECOG PS, the Practicing group tended to detect cancer cachexia and initiate nutritional and exercise interventions in earlier phases and in patients with a better status, although no statistically significant differences were found between the groups. Moreover, most of the pharmacists in both groups considered that neither detecting cachexia nor initiating nutritional or physical interventions had any relation to the life expectancy of patients. 

**Figure 2:**
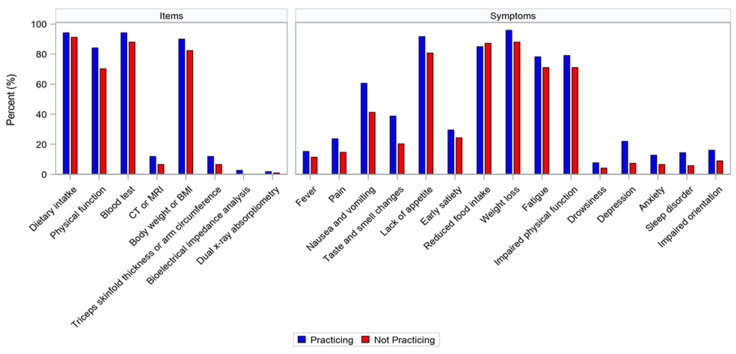
Items and symptoms used in the assessment of cancer cachexia. Percentages for each item and symptom in the group practicing multimodal care and the group not practicing multimodal care are shown in the bar graph. CT, computed tomography; MRI, magnetic resonance imaging.

Frequencies of using guidelines, items, and symptoms in the assessment of cancer cachexia are presented in Table 4. The Practicing group used guidelines, items, and symptoms more frequently to detect cancer cachexia than the Not practicing group, with significant differences. Percentages for each item and symptom in the Practicing and Not practicing groups are shown in Figure 2. The percentages for all items and symptoms, except for reduced food intake, tended to be higher in the Practicing group. 

Results of the multivariate logistic regression analysis, presented in Table 5, show that the most significant factor was the regular provision of care for patients with advanced cancer (odds ratio [OR] 2.07, 95% confidence interval [CI] 1.10–3.92, *p*-value 0.025). In addition, the number of advanced cancer patients per month (i.e., 50–99 patients/month) and the number of symptoms used in the assessment of cancer cachexia tended to be more different than other factors (OR 3.31, 95% CI 0.92–11.93, *p*-value 0.068; OR 1.12, 95% CI 0.99–1.26, *p*-value 0.078, respectively).

## 4. Discussion

This was the first study to examine the knowledge, perception, and practice of cancer cachexia care and investigate the factors that influence the practice of multimodal care for cancer cachexia in pharmacists engaging in cancer care. The study demonstrated that pharmacists with the perception of practicing multimodal cachexia care had more knowledge about cancer cachexia and used guidelines, items, and symptoms more often in the assessment of cancer cachexia than those without the perception. Furthermore, the findings revealed that the regular provision of care for patients with advanced cancer was strongly associated with the practice of multimodal care for cancer cachexia. 

The results suggested that it is very important for pharmacists, as well as physicians and nurses, to be regularly and directly involved in the care of patients with advanced cancer. Pharmacists who practice as members of a multidisciplinary team can propose optimal drug therapy from a pharmacological point of view, considering the rapidly changing conditions of patients and their cancer treatment status [9]. Additionally, the pharmacist’s knowledge and skills may naturally increase through comprehensive and collaborative assessment and management of the patient with other members of the team, potentially enhancing the confidence levels of each party [15,16,17,18].

The results of this study also revealed that pharmacists do not have sufficient opportunities to receive training or learn about the pathophysiology and management of cancer cachexia. However, understanding cancer cachexia is essential for pharmacists to provide multimodal care to patients with advanced cancer [15,16,17,18]. In addition, since the potential effectiveness of some drugs for cancer cachexia has been reported recently [5,6,7], opportunities to provide physicians with direct suggestions for prescribing such drugs may be increasing. Therefore, the development of training programs for pharmacists may support the practice of multidisciplinary care based on the appropriate knowledge and skills. 

Pharmacists are pharmacological experts who are required to have a high level of specialized knowledge and technical literacy. Academic detailing, a new approach to drug information provision that actively disseminates comparative drug information based on basic science and clinical evidence, is a role that has been performed by pharmacists since the 1990s [21]. In recent years, drug information has become increasingly multidisciplinary and specialized. For example, pharmacists with expertise in various fields, such as oncology, palliative care, nutritional care, and infection control, are in demand. Such pharmacists should be highly motivated to improve their relevant knowledge and skills so that they can provide the best pharmacotherapy in their respective clinical settings.

This study has several strengths and limitations. The large scale of the study and moderate response rate despite the COVID-19 pandemic were considered strengths. However, the survey was conducted in one country, and thus the findings cannot be generalized to other countries. Moreover, the results showed that the group practicing multimodal cachexia care was more likely to evaluate items and symptoms. However, the provision of routine care to patients may be influenced not only by knowledge but also by environmental matters. Since participants of the study belonged to cancer-designated hospitals, they might be more aware of cancer care than the general hospital population. Furthermore, the nine components of multimodal cancer cachexia care used in this study have not been validated, although the ability of the nine components to assess the practice of multimodal cancer cachexia care in multiple professions involved in cancer care was suggested in a previous study [18]. In addition, common drugs available for cancer or cancer cachexia were missing in the questionnaire because the survey did not target only pharmacists. Further research is needed on the development of multimodal care that considers the characteristics of pharmacists from the viewpoint of pharmacology. 

## 5. Conclusion

The extensive knowledge on cancer cachexia and the regular provision of care to patients with advanced cancer were significantly associated with the practice of multimodal care for cancer cachexia among pharmacists engaging in cancer care. Specific training programs on the management of cancer cachexia must be developed for pharmacists to encourage them to be aware of cancer cachexia, utilize evidence-based clinical practice guidelines on cancer cachexia, and regularly provide care to patients with advanced cancer. Pharmacists can play a key role in a multidisciplinary care team, and continuous pharmacological support is needed for supportive and palliative care for cancer cachexia. 

## Figures and Tables

**Table 1:** Participant characteristics.

	Total	Group Practicing Multimodal Cachexia Care	Group Not Practicing Multimodal Cachexia Care	*p*-Value
	N = 243	N = 119	N = 124	
Age, years	40.0 ± 7.6	40.1 ± 7.1	40.0 ± 8.2	0.945
Sex, male	136 (56.0%)	68 (57.1%)	68 (54.8%)	0.710
Practicing experience				0.072
3–4 years	11 (4.5%)	5 (4.2%)	6 (4.8%)	
5–9 years	60 (24.7%)	24 (20.2%)	36 (29.0%)	
10–19 years	106 (43.6%)	62 (52.1%)	44 (35.5%)	
20 or more years	64 (26.3%)	27 (22.7%)	37 (29.8%)	
Practicing experience in cancer care				0.200
2 or fewer years	18 (7.4%)	9 (7.6%)	9 (7.3%)	
3–4 years	26 (10.7%)	8 (6.7%)	18 (14.5%)	
5–9 years	76 (31.3%)	35 (29.4%)	41 (33.1%)	
10–19 years	108 (44.4%)	58 (48.7%)	50 (40.3%)	
20 or more years	14 (5.8%)	9 (7.6%)	5 (4.0%)	
Number of patients with advanced cancer/month				0.004
1–9	36 (14.8%)	10 (8.4%)	26 (21.0%)	
10–19	76 (31.3%)	33 (27.7%)	43 (34.7%)	
20–49	88 (36.2%)	48 (40.3%)	40 (32.3%)	
50–99	27 (11.1%)	19 (16.0%)	8 (6.5%)	
100 or more	10 (4.1%)	7 (5.9%)	3 (2.4%)	
Primary area of practice				0.497
Palliative care	96 (39.5%)	45 (37.8%)	51 (41.1%)	
Cancer treatment	124 (51.0%)	65 (54.6%)	59 (47.6%)	
Others	20 (8.2%)	8 (6.7%)	12 (9.7%)	
Frequency of caring for patients with advanced cancer				< 0.001
Regularly	145 (59.7%)	88 (73.9%)	57 (46.0%)	
Only the first time or when needed	94 (38.7%)	30 (25.2%)	64 (51.6%)	
Receiving training programs on the management of cancer cachexia, yes	18 (7.4%)	14 (11.8%)	4 (3.2%)	0.012

Values are n (%) or the mean ± standard deviation.

**Table 2:** Knowledge and utilization of the international definition and clinical practice guidelines.

	Group Practicing Multimodal Cachexia Care	Group Not Practicing Multimodal Cachexia Care	*p*-Value
	N = 119	N = 124	
International definition of cancer cachexia			
Knowledge, yes	73 (61.3%)	38 (30.6%)	<0.001
Application, yes	49 (41.2%)	18 (14.5%)	<0.001
ASCO guidelines			
Knowledge, yes	49 (41.2%)	27 (21.8%)	0.001
Application, yes	16 (13.4%)	7 (5.6%)	0.034
ESMO guidelines			
Knowledge, yes	27 (22.7%)	10 (8.1%)	0.002
Application, yes	7 (5.9%)	3 (2.4%)	0.288
ESPEN guidelines			
Knowledge, yes	33 (27.7%)	11 (8.9%)	<0.001
Application, yes	16 (13.4%)	2 (1.6%)	<0.001

Values are n (%). ASCO, American Society of Clinical Oncology; ESMO, European Society for Medical Oncology; ESPEN, European Society of Clinical Nutrition and Metabolism.

**Table 3:** Perception and knowledge of cancer cachexia.

	Group Practicing Multimodal Cachexia Care	Group Not Practicing Multimodal Cachexia Care	*p*-Value
	N = 119	N = 124	
Weight loss rate			
Status considered as cancer cachexia			0.104
<2%	0 (0.0%)	0 (0.0%)	
≥2%	1 (0.8%)	0 (0.0%)	
≥5%	90 (75.6%)	81 (65.3%)	
≥10%	27 (22.7%)	35 (28.2%)	
≥15%	0 (0.0%)	3 (2.4%)	
≥20%	1 (0.8%)	4 (3.2%)	
Initiation of nutritional and physical interventions			0.092
<2%	4 (3.4%)	3 (2.4%)	
≥2%	27 (22.7%)	25 (20.2%)	
≥5%	72 (60.5%)	63 (50.8%)	
≥10%	15 (12.6%)	29 (23.4%)	
≥15%	0 (0.0%)	2 (1.6%)	
≥20%	0 (0.0%)	2 (1.6%)	
ECOG PS			
Status considered as cancer cachexia			0.648
PS 0	2 (1.7%)	3 (2.4%)	
PS 1	16 (13.4%)	15 (12.1%)	
PS 2	70 (58.8%)	64 (51.6%)	
PS 3	29 (24.4%)	40 (32.3%)	
PS 4	2 (1.7%)	1 (0.8%)	
Initiation of nutritional and physical interventions			0.817
PS 0	10 (8.4%)	8 (6.5%)	
PS 1	47 (39.5%)	51 (41.1%)	
PS 2	55 (46.2%)	61 (49.2%)	
PS 3	6 (5.0%)	4 (3.2%)	
PS 4	0 (0.0%)	0 (0.0%)	
Life expectancy			
Status considered as cancer cachexia			0.849
<1 week	0 (0.0%)	0 (0.0%)	
<2 weeks	0 (0.0%)	1 (0.8%)	
<1 month	10 (8.4%)	12 (9.7%)	
<3 months	25 (21.0%)	24 (19.4%)	
<6 months	15 (12.6%)	20 (16.1%)	
Unrelated	69 (58.0%)	67 (54.0%)	
Initiation of nutritional and physical interventions			0.286
<1 week	0 (0.0%)	0 (0.0%)	
<2 weeks	0 (0.0%)	0 (0.0%)	
<1 month	1 (0.8%)	6 (4.8%)	
<3 months	6 (5.0%)	6 (4.8%)	
<6 months	14 (11.8%)	11 (8.9%)	
Unrelated	98 (82.4%)	100 (80.6%)	

Values are n (%). ECOG PS, Eastern Cooperative Oncology Group performance status.

**Table 4:** Guidelines, items, and symptoms used in the assessment of cancer cachexia.

	Group Practicing Multimodal Cachexia Care	Group Not Practicing Multimodal Cachexia Care	*p*-Value
	N = 119	N = 124	
Guidelines			
Number of guidelines used			< 0.001
Mean (SD)	0.75 (0.94)	0.24 (0.60)	
Median (IQR)	0.0 (0.0–1.0)	0.0 (0.0–0.0)	
Range	0.0–4.0	0.0–4.0	
Items			
Number of items used			< 0.001
Mean (SD)	3.91 (0.82)	3.49 (1.02)	
Median (IQR)	4.0 (4.0–4.0)	4.0 (3.0–4.0)	
Range	1.0–6.0	1.0–6.0	
Symptoms			
Number of symptoms used			< 0.001
Mean (SD)	6.69 (2.88)	5.41 (2.85)	
Median (IQR)	6.0 (5.0–8.0)	5.0 (4.0–7.0)	
Range	0.0–15.0	0.0–15.0	

SD, standard deviation; IQR, interquartile range.

**Table 5:** Multivariate logistic regression analysis of multimodal care for cancer cachexia.

	Odds Ratio (95% CI)	*p*-Value
Participant characteristics		
Number of patients with advanced cancer/month (ref = 1–9)		
10–19	1.31 (0.49–3.47)	0.587
20–49	1.65 (0.62–4.35)	0.315
50–99	3.31 (0.92–11.93)	0.068
100 or more	2.93 (0.56–15.42)	0.205
Frequency of caring for patients with advanced cancer (ref = only the first time or when needed)		
Regularly	2.07 (1.10–3.92)	0.025
Participation in training programs on the management of cancer cachexia (ref = no)		
Yes	2.76 (0.78–9.83)	0.116
International definition used in the assessment of cancer cachexia		
Application of the international definition of cancer cachexia (ref = no)		
Yes	1.81 (0.64–5.10)	0.265
Guidelines, items, and symptoms used in the assessment of cancer cachexia		
Number of guidelines used	1.55 (0.83–2.92)	0.172
Number of items used	1.17 (0.80–1.72)	0.421
Number of symptoms used	1.12 (0.99–1.26)	0.078

The number of patients with advanced cancer/month; frequency of caring for patients with advanced cancer; participation in training programs on management of cancer cachexia; application of the international definition of cancer cachexia; and the numbers of guidelines, items, and symptoms used in the assessment of cancer cachexia were included in the multivariate analysis. CI, confidence interval.

## Data Availability

The original contributions presented in this study are included in the article. Further inquiries can be directed to the corresponding author.
